# Time to childbirth and assisted reproductive treatment in women with congenital heart disease

**DOI:** 10.1136/openhrt-2023-002591

**Published:** 2024-03-13

**Authors:** Sara Jonsson, Inger Sundström-Poromaa, Bengt Johansson, Jenny Alenius Dahlqvist, Christina Christersson, Mikael Dellborg, Alexandra Trzebiatowska-Krzynska, Peder Sörensson, Ulf Thilén, Anna-Karin Wikström, Annika Bay

**Affiliations:** 1 Department of Public Health and Clinical Medicine, Umeå University, Umeå, Sweden; 2 Department of Women’s and Children’s Health, Uppsala University, Uppsala, Sweden; 3 Department of Surgical and Perioperative Sciences, Umeå University, Umeå, Sweden; 4 Department of Clinical Sciences, Umeå University, Umeå, Sweden; 5 Department of Medical Sciences, Cardiology, Uppsala University, Uppsala, Sweden; 6 Department of Clinical and Molecular Medicine, Sahlgrenska Academy, University of Gothenburg, Goteborg, Sweden; 7 Department of Health, Medicine and Caring Sciences, Linköping University, Linköping, Sweden; 8 Department of Medicine, Solna, Karolinska Institutet, Stockholm, Sweden; 9 Department of Cardiology, Karolinska University Hospital, Stockholm, Sweden; 10 Department of Clinical Sciences, Lund University, Lund, Sweden; 11 Department of Nursing, Umeå University, Umeå, Sweden

**Keywords:** heart defects, congenital, pregnancy, epidemiology

## Abstract

**Objective:**

To investigate the time to first childbirth and to compare the prevalence of assisted reproductive treatment (ART) in women with congenital heart disease (CHD) compared with women without CHD.

**Methods:**

All women in the national register for CHD who had a registered first childbirth in the Swedish Pregnancy Register between 2014 and 2019 were identified. These individuals (cases) were matched by birth year and municipality to women without CHD (controls) in a 1:5 ratio. The time from the 18th birthday to the first childbirth and the prevalence of ART was compared between cases and controls.

**Results:**

830 first childbirths in cases were identified and compared with 4137 controls. Cases were slightly older at the time for first childbirth (28.9 vs 28.5 years, p=0.04) and ART was more common (6.1% vs 4.0%, p<0.01) compared with controls. There were no differences in ART when stratifying for the complexity of CHD. For all women, higher age was associated with ART treatment (OR 1.24, 95% CI 1.20 to 1.28).

**Conclusions:**

Women with and without CHD who gave birth to a first child did so at similar ages. ART was more common in women with CHD, but disease severity did not influence the need for ART. Age was an important risk factor for ART also in women with CHD and should be considered in consultations with these patients.

**WHAT IS ALREADY KNOWN ON THIS TOPIC**

**WHAT THIS STUDY ADDS**

- Our study showed that women with and without CHD gave birth to their first child at a similar age.
- Furthermore, we found that treatment with ART was more common in women with CHD.

**HOW THIS STUDY MIGHT AFFECT RESEARCH, PRACTICE OR POLICY**

- The results of this study showed that assisted reproductive techniques like ART are more common among women with CHD and that there is an increased need for ART with increasing age. This is important information that can be used in preconception counselling.

## Introduction

 The long-term survival of children with congenital heart disease (CHD) has increased. As a result, more women with CHD reach adulthood and childbearing age.^1 2^ Family planning and pregnancy is therefore an important issue for many of these women. Furthermore, motherhood is often an important contributor to quality of life. Several studies have provided knowledge about maternal and neonatal complications during pregnancy in women with CHD^3^,^7^; however, few studies have examined fertility in this population. Women with CHD, especially complex and cyanotic CHD, have a later menarche and more frequently menstrual disorders.^8 9^ In addition, there are indications that infertility and menstrual cycle disorders are more prevalent among some women with CHD.^10^ On the other hand, a recent register study indicated that men and women with mild to moderate CHD have the same risk of infertility as the reference population.^11^ More knowledge is needed in this field to provide adequate counselling to the growing number of women with CHD planning pregnancy.

Our primary aim was to investigate the time to first childbirth and the prevalence of assisted reproductive treatment (ART) in women with CHD compared with age-matched women without CHD. A secondary aim was to identify factors associated with the prevalence of ART in these women.

## Methods

The present study is a register study created by linking two Swedish national registers. The Swedish Register of Congenital Heart Disease (SWEDCON) contained approximately 16 000 adult individuals in 2019, of which approximately 50% were women.^12^ Examples of data in the register are social and demographic variables (eg, age, marital status, housing, highest education and employment status), medical data (eg, diagnosis, medication, catheterisation or catheter interventions, type of surgery and need of pacemaker), physiological data (eg, ECG and echocardiogram), general symptoms and functional class scored by trained healthcare professionals according to the New York Heart Association classification system.

The Swedish Pregnancy Register contains data on pregnancy resulting in a childbirth >22 weeks of gestation, starting at the first visit at antenatal care and ending at the follow-up visit at antenatal care, that typically occurs 8–16 weeks post partum. The register started in its current form in 2014 with automatic data transfer from medical charts and covers >90% of all births in Sweden. The register includes demographic (eg, educational level, body mass index (BMI), country of birth), obstetric (eg, previous pregnancies) reproductive (eg, ART and time to achieve pregnancy) and maternal health data; also included are prenatal diagnostics, pregnancy outcomes and complications at the delivery for the mother and the newborn.^13^ ART includes in vitro fertilisation and a few cases of intracytoplasmic sperm injection (ICSI). The two registers were linked using the unique civil registration number that is assigned to Swedish residents at birth or on immigration.^14^

### Study population

Included in the study were women with CHD registered in SWEDCON and in the Swedish Pregnancy Register who gave birth between 2014 and 2019.

Only women with congenital structural heart disease were included. Isolated persistent ductus arteriosus closed early in life, isolated foramen ovale and irrelevant diagnoses/investigations such as isolated arrhythmia and ‘evaluation of physiologic murmur’ were excluded. 830 primiparous women with structural CHD were identified. The women were categorised into three groups based on the complexity of CHD—mild (n=624), moderate (n=173) and severe (n=33), according to the European Society of Cardiology guidelines.^15^ Continuous variables were dichotomised, BMI<25 and *≥25* and level of education ≤12 and >12 years (in Sweden, university/college level usually starts after 12 years of education).

**Figure 1:**
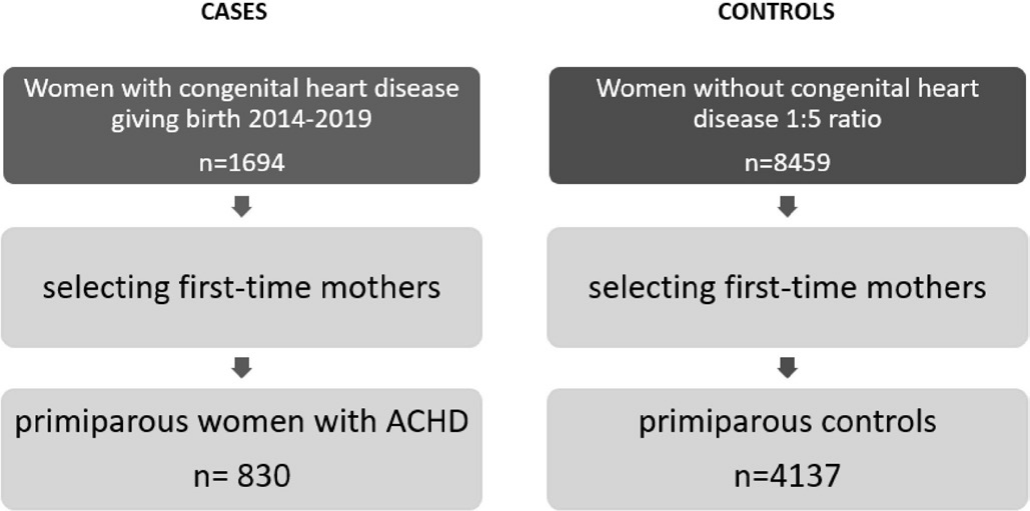
Flow chart of study population. ACHD, adult congenital heart disease.

For each woman with CHD (cases), five women without CHD born in the same year and living in the same municipality were randomly drawn from the Swedish Pregnancy Register (controls). This resulted in 4137 controls since it was not possible to find five matching controls if the municipality was too small (figure 1).

### Statistical analysis

The Statistical Package for Social Sciences (SPSS V.27) (IBM) was used for statistical analyses. Student’s t-test, χ² test and Fisher’s exact test were used for comparing means and ratios. Kaplan-Meier curves were used to estimate the time to childbirth, and comparisons between groups were made using the log-rank test. Independent variables were assessed using univariable logistic regression. Multivariable models were constructed that included variables with a p value <0.10 in the univariable analysis. The model was assessed using a manual backward method, and the number of cases and multicollinearity were considered in each step. ORs are presented with 95% CIs. The null hypothesis was rejected on p values <0.05. Cases and controls with missing data were excluded from analyses.

Patients or the public were not involved in the design, conduct, reporting or dissemination plans of our research.

## Results

830 pregnancies were identified in women with CHD during 2014–2019 and were compared with women without CHD (n=4137) (figure 1). Characteristics of the study population are shown in tables1  2.

**Table 1:** Overview of the baseline characteristics among women with CHD and controls

		Total populationn=4967	Controls(n=4137)	Women with CHD(n=830)	P value
Age, years	Mean (±SD)	28.6 (4.6)	28.5 (4.5)	28.9 (4.7)	0.04*
Simple lesions (n=624)				28.7 (4.7)	0.16†
Moderate/severe lesions (n=206)				29.2 (4.6)	
ART (n=4967), yes	n (%)	218	167 (4.0)	51 (6.1)	**0.007** *
Simple lesions		624		39 (6.3)	0.83‡
Moderate/severe lesions		206		12 (5.8)	
Previous pregnancies	n (%)				0.93
One previous		980 (19.7)	816 (19.7)	164 (19.8)	
Two or more previous		976 (19.6)	809 (19.6)	167 (20.1)	
Education (n=4540)	n (%)				0.29
≤12 years			2014 (53.2)	384 (51.1)	
>12 years			1774 (46.8)	368 (48.9)	
BMI, kg/m^2^ (n=4631)	Mean (±SD)	24.5 (4.5)	24.5 (4.6)	24.4 (4.5)	0.62

* P value for comparison between cases and controls.

† P value for comparison between age in women with simple versus moderate/severe congenital heart disease. Bold p values indicate statistical significance, p≤0.05.

‡ P value for comparison between groups with different complexity of CHD.

ART, assisted reproductive treatment; BMI, body mass index; CHD, congenital heart disease.

**Table 2:** Overview of congenital heart diagnoses

Diagnosisn (%)	Totaln=830 (%)	ART, yesn=51 (%)
VSD	247 (29.8)	18 (35.3)
ASD	161 (19.4)	8 (15.7)
Pulmonary stenosis	81 (9.8)	2 (3.9)
Truncus arteriosus	2 (0.2)	0
Marfan	16 (1.9)	3 (5.9)
Tetralogy of Fallot	27 (3.3)	1 (2.0)
TGA, arterial switch	10 (1.2)	1 (2.0)
Pulmonal atresia with IVS	1 (0.1)	0
Pulmonal atresia with VSD	6 (0.7)	0
Double outlet right ventricle	4 (0.5)	0
Aortic valve disease	103 (12.4)	7 (13.7)
Ebstein’s anomaly	7 (0.8)	0
AVSD	10 (1.2)	0
Coarctation of the aorta	64 (7.7)	6 (11.8)
Fontan circulation	6 (0.7)	0
Congenitally corrected TGA	2 (0.2)	0
TGA, atrial switch	9 (1.2)	1 (2.0)
Miscellaneous	73 (8.8)	4* (7.8)

* Three partially anomalous pulmonary vein drainage, one tricuspid insufficiency and one ballooning mitral valve.

ART, assisted reproductive treatment; ASD, atrial septal defect; AVSD, atrioventricular septal defect; IVS, intact ventricular septum; TGA, transposition of the great arteries; VSD, ventricular septal defect.

**Figure 2:**
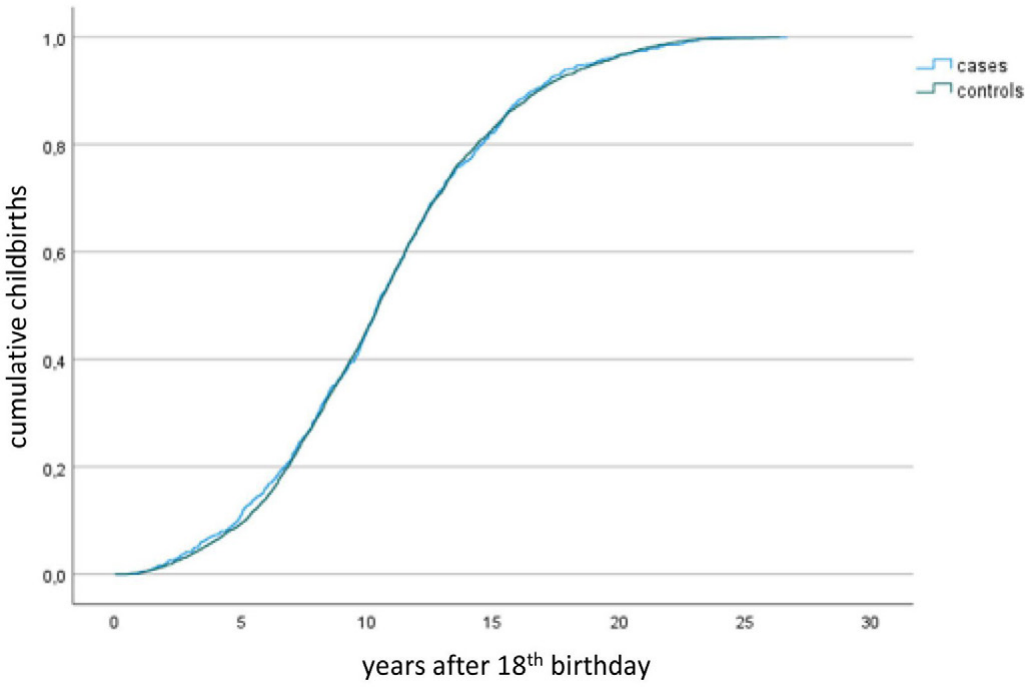
Kaplan-Meier curves showing time to first childbirth from the 18th birthday in years. There were no differences between cases and controls (log-rank statistics, p=0.96).

Women with CHD were slightly older at the time for first childbirth (28.9±4.7 vs 28.5±4.5 years, p=0.04). There were no differences in age at first childbirth when stratifying for the complexity of CHD. Educational level, BMI and number of previous pregnancies (miscarriages, extrauterine pregnancies, induced abortions) were similar in both groups (table 1). The time to first childbirth and pattern of childbirth was similar in cases and controls (p=0.96) (figure 2).

Assisted reproduction through ART was more common in women with CHD giving birth to their first child compared with controls (6.1% vs 4.0%, p=0.007) (table 1). The number of ICSIs was 5 (0.7%) within the CHD group and 13 (0.3%) within the control group.

Among women with CHD, those treated with ART were older when giving birth to their first child and had a higher educational level (table 3).

**Table 3:** Overview of ART in women with CHD

		ART, yesn=218 (%)	ART, non=4749 (%)	P value
Age, years	mean (±SD)	33 (4.3)	28.4 (4.5)	**<0.001**
BMI (kg/m^2^)	n (%)			0.94
<25		131 (63.0)	2812 (63.5)	
≥25		77 (37.0)	1611 (36.4)	
Education	n (%)			**<0.001**
≤12 years		74 (36.5)	2324 (53.6)	
>12 years		129 (63.5)	2013 (46.4)	
CHD diagnosis	n (%)	51	779	**0.007**
Simple		39 (76.5)	585 (75.1)	0.82
Moderate/severe		12 (23.5)	194 (24.9)	

Bold p values indicate statistical significance, p≤0.05.

Moderate/severe includes 1 severe case.

ART, assisted reproductive treatment; BMI, body mass index; CHD, congenital heart disease.

In women with and without CHD, univariable logistic regression showed an association between age and ART, as well as educational level and ART (table 4). When controlled for age, the association between educational level and ART disappeared. Among all women, older age was associated with ART (OR 1.24, 95% CI 1.20 to 1.28).

**Table 4:** Factors associated with ART in women with and without CHD

	OR	95% CI	P value
Age	1.23	1.20 to 1.27	**<0.001**
BMI	0.99	0.96 to 1.02	0.57
Education >12 years	1.97	1.47 to 2.65	**<0.001**
CHD diagnosis	1.59	1.15 to 2.20	0.05
Complexity of CHD, moderate/severe*	0.96	0.49 to 1.88	0.91

Data assessed with univariable logistic regression.

Bold p values indicate statistical significance, p≤0.05.

* Indicates that this regression was performed only for women with CHD.

ART, assisted reproductive treatment; BMI, body mass index; CHD, congenital heart disease.

## Discussion

Among women with CHD who achieved a first-time birth, the time to pregnancy was similar compared with women without CHD. We also showed that assisted reproductive techniques like ART are more common among women with CHD. Age is, as in all women, a major factor affecting fertility and a risk factor associated with ART.^16 17^ Our results are encouraging for women with CHD who plan for a family, and highlight the higher risk of needing ART especially for those at a relatively older age.

Infertility in a population is difficult to measure because population-based registers have no information on which couples (or women) are attempting a pregnancy at a given time. In our cohort, we show that the time from the 18th birthday until the birth of the first child is similar in women with and without CHD, at least among those who achieve a childbirth. There was no difference in age at first delivery detected when stratifying for complexity of the heart lesion. It may be argued that women with the most complex heart lesions have been advised against pregnancy or have chosen not to become pregnant^18^; but still individuals in our study follow the same pattern irrespective of CHD and its complexity.

Treatment with ART was more common in women with CHD. In both cases and controls, older age was associated with ART. Higher educational level was associated with ART, however not when controlled for age. Therefore, educational level is most likely a surrogate marker for age and not itself associated with ART. It is not clear why ART is more common in women with CHD but it may be speculated that a more generally affected health is a major contributing factor, resulting in subfertility.^19^ Furthermore, women with CHD, who are already within the healthcare system, may be more likely to seek medical attention for infertility. In this context, it is important to note that these women in the present study gave birth to a child after ART, and we do not have data on those with ART without a successful pregnancy. This is, however, also valid for the women without CHD.

In CHD, complications tend to accumulate with increasing age. This is also true for conditions relevant for pregnancy and delivery.^20^,^22^ Important examples are valve replacement with mechanical valve protheses and need for warfarin treatment, heart failure with medication that puts the fetus at risk and arrhythmias that may be triggered during pregnancy. These examples, together with an increased need for ART with increasing age, make it relevant to advise women with CHD to have their children earlier. It is also important to note that the risk for ART associated with higher education as seen in our univariable analysis was cancelled when controlled for age. This shows that the need for ART remains associated with age and that higher education only indicates an association to older age and does not in itself point at a risk.

In conclusion, women with and without CHD who gave birth to a first child did so at similar ages. ART was more common in women with CHD, but disease severity did not influence the need for ART. Age was an important risk factor for ART also in women with CHD and should be considered in consultations with these patients.

### Limitations

This is a register study, limited to the data within the register. As in all observational studies, causality cannot be determined. One study limitation is that the data only cover women who have given birth to a child. We do not have data on women who tried to become pregnant during the study period but did not succeed. Therefore, information is missing about miscarriages and fertility treatment not resulting in a childbirth. This is, however, true for both cases and controls. Infertility also involves the partner, but if so, often results in ART. Here, we do not have data on the partner but can still compare cases and women without CHD.

It cannot be excluded that some women with complex CHD were advised against pregnancy. In such case, they would only represent a minority of the population of women with CHD; therefore, it is unlikely that they would change the main results of this study.

## Data Availability

Data are available upon reasonable request.
